# Trauma Exposure and Prolonged Grief Disorder Among Persons Receiving Community Mental Health Services: Rates and Correlates

**DOI:** 10.3389/fpsyt.2021.760837

**Published:** 2022-02-04

**Authors:** Weili Lu, Philip T. Yanos, William R. Waynor, Carol E. Gao, Carolyn Bazan, Giovanna Giacobbe, Kenneth Gill, Deanna Bullock, Holly G. Prigerson

**Affiliations:** ^1^Department of Psychiatric Rehabilitation and Counseling Professions, Rutgers University, New Brunswick, NJ, United States; ^2^John Jay College, City University of New York, New York City, NY, United States; ^3^Department of Medicine, Center for Research on End of Life Care, Cornell University, White Plains, NY, United States

**Keywords:** serious mental illness (SMI), community mental health services, PTSD, Prolonged Grief Disorder (PGD), trauma

## Abstract

**Background:**

Persons with serious mental illnesses (SMIs) are at increased risk for exposure to trauma and posttraumatic stress disorder (PTSD). Prolonged Grief Disorder (PGD) may also impact this population but has been seldom studied.

**Aims:**

The present study investigated the rate of both PTSD and PGD among clients receiving community mental health services, and the clinical correlates of co-occurring PTSD/PGD.

**Methods:**

Trauma history, PTSD and PGD were assessed among 536 individuals receiving community mental health services (Study 1). A subsample of 127 individuals from Study 1 who met DSM-5 criteria for PTSD based on diagnostic interview completed measures of psychiatric symptoms (Study 2).

**Results:**

In Study 1, 92.4% of participants receiving community mental health services had experienced a traumatic event, 49.6% met criteria for probable and provisional PTSD, 14.7% scored positive for probable PGD, and 11.9% met criteria for probable and provisional PTSD as well as probable PGD. In Study 2, participants meeting diagnostic DSM-5 criteria for PTSD and probable PGD had more self-reported PTSD symptoms, but did not differ on other outcomes.

**Conclusions:**

Findings highlight the need for trauma informed services including grief counseling for persons with SMI.

## Introduction

The lifetime prevalence of PTSD ranges from 5 to 10%, according to studies of PTSD conducted among the general population of the United States and Europe (1, 2), with women are more likely to develop PTSD than men (3). Serious mental illnesses (SMIs) are mental, behavioral, or emotional disorders that result in serious functional impairment, i.e., that affect an individual's ability to perform major life activities such as working, maintaining social relationships, or taking care of oneself (4) (the most common SMIs include, but are not limited to, schizophrenia, schizoaffective disorder, bipolar disorder, and major depression). Research supports that trauma and SMI are intertwined in two ways: (1) individuals can develop SMI after being exposed to trauma in their childhood (5, 6), and (2) individuals who develop SMI also have a higher risk of experiencing violent victimization (7). As a result, exposure to multiple traumas is nearly universal for individuals with SMI (8), and people with SMI have higher rates trauma exposure than individuals without SMI (9–12). Further, the prevalence of PTSD has been reported as 25–48% among people with SMI (9), almost ten times that of the general population (2). Research indicates that co-occurring PTSD can worsen the course of SMI, leading to more severe symptoms, (including psychosis, anxiety, and depression), psychosocial impairment, lower quality of life, poorer physical health and greater use of psychiatric and emergency services (13–15). In addition, studies have shown gender difference on cumulative impact of trauma on psychosis: childhood physical abuse was experienced more frequently by males with psychosis, while childhood sexual abuse and recent traumatic life experiences were more prevalent in females with psychosis (16). All together, SMI patients exposed to childhood trauma had poor clinical outcomes compared to those not exposed to childhood trauma, such as increased negative cognitions as well as dysfunctional metacognitive beliefs, and more severe psychiatric symptoms (16–19).

Consistent with findings among the general population (20), sudden death was reported to be the leading cause of PTSD in a study among persons with SMI (10) and was experienced by nearly 80% of the participants. The high frequency of sudden death of a loved one, including violent death in this sample of clients with SMI, and its association with PTSD, raises the question of the related syndrome of Prolonged Grief Disorder [PGD; (21)], which can follow the sudden, unexpected death of a loved one (22). PGD is marked by disabling separation, distress and grief-specific symptoms, such as yearning for the deceased, difficulties accepting the death, and difficulties moving on in life, that are distinct from both PTSD and depression (23, 24). Prevalence rates of PGD are estimated to range from 7 to 20% after a person experiences bereavement (24, 25). Persons with PGD are more likely to develop health complications, die by suicide, and be diagnosed with an affective or anxiety disorder (26, 27). As research indicates that the unexpected death of a loved one is the most commonly reported trauma among participants receiving public mental health treatment (10, 11), it is plausible that the combination of trauma and PGD may contribute to multi-morbidity for persons with SMI (28, 29). The notion of multi-morbidity, or two or more chronic conditions, is a relatively new conceptualization of medical complexity in medicine, including psychiatry, and is associated with increased disability, mortality, and decreased quality of life (28, 30). SMI clients with PTSD and PGD may present a subset of multi-morbidity, which may lead to increased disability (31).

Despite significant overlap between PTSD and PGD (32, 33), to date, there has been little research examining PTSD and PGD and associated outcomes among SMI clients. The aims of the present study were: (1) to assess the rates of both PTSD and PGD in one sample of SMI clients (Study 1); (2) to report on the clinical correlates of PTSD and PGD (Study 2). We hypothesized that, consistent with multi-morbidity theory (28, 30), SMI clients with PTSD and PGD would have worse symptoms and more impairment compared to those with PTSD only. Toward this end, the present study reports findings from a multi-site comprehensive screening of SMI clients (Study 1). We also report on the clinical and functional correlates of combined PTSD and PGD among a smaller sample of individuals found to meet criteria DSM-5 criteria for PTSD (Study 2).

## Methods

Findings reported here were drawn from a larger randomized control trial (RCT) comparing a 12-week group cognitive behavioral treatment (CBT) for PTSD program with treatment as usual (TAU) in community mental health programs, serving clients with SMI, operated in three Northeastern states (Lu et al., unpublished^1^). The study protocol, consent, and all study-related materials were reviewed and approved by the university Institutional Review Board. Study participants were people with SMI (schizophrenia, major depression, bipolar disorder, etc.) receiving vocational services at 10 community mental health programs. The study sites were located in urban, suburban and rural communities. Additionally, the community mental health programs were all part of larger mental health agencies that provided an array of services including; pharmacological treatment, psychological treatment, supportive housing, partial care, medication management, counseling, peer support, assertive community treatment, and other case management programs. In order to facilitate referrals for the RCT (Study 2), trauma history and PTSD screening were implemented at these sites (Study 1). Participants who were screened for PTSD for the RCT comprised Study 1. Study 2 consisted of those who met criteria for PTSD and were enrolled in the RCT.

In study 1, program and study staff were trained to conduct PTSD and PGD screening and coordinated to choose dates for screenings at their respective programs. Staff then notified the program clients of the opportunity to be screened for PTSD, and the dates the study staff would conduct the screenings at the agency. Agency staff posted flyers in the office as well as made personal calls to clients informing them of the day of the screening (note that the invitation to participate was open to all clients at the recruitment sites). Individuals who were interested came to the program on the day of the screening and met with study personnel who explained the screening process. If the individual agreed, study personnel and staff conducted a comprehensive screening of trauma exposure and PTSD and PGD symptoms. Participants first completed a trauma screening measure, and if they indicated “yes” on sudden death of a loved one were asked to complete PGD measure. Participants who agreed then completed a permission to contact form, Authorization of Release of Protected Health Information form, and a consent form to have the results of the screening provided to the research team. Additionally, an eligibility checklist was filled out by participants who included self-reported diagnoses. Sex was recorded as noted by the interviewers. Participants were paid $10 for completing the screening.

Upon completion of the trauma screening in Study 1, participants were then asked if they were willing to have their screening data provided to the research team for possible participation in a treatment study of CBT for PTSD (Study 2). Participants indicated whether they would be interested in being contacted by the research team if they met preliminary eligibility criteria for the study. For Study 2, inclusion criteria were the following: (1) age ≥ 18; (2) currently receiving SE services within the past 24 months; (3) history of treatment for mental illness; (4) current diagnosis of PTSD; (5) no current diagnosis of alcohol or drug dependence as described in chart; (6) no hospitalization or suicide attempt in the past 2 months; and (7) willingness to provide informed consent to participate in the study. Potentially eligible and interested clients were contacted by a team member, who described the study and obtained informed consent. Once consent was obtained, the completion of a baseline interview would confirm the eligibility of participation. Participants were paid $30 for the completion of the baseline interview.

### Participants

In Study 1, 536 persons produced usable screening assessments, characterized by having more than half of data points recorded. Table 1 summarizes demographic characteristics. Participants were closely split by gender, nearly half of participants identified as African American, and participants tended to be in their late 40's (*M* = 47.23, SD = 12.91). Three hundred and thirty-four (62.3%) participants reported their psychiatric diagnoses on the Eligibility Checklist. Among these, the most common self-reported diagnoses were Depressive Disorders (34.1%), Bipolar Disorders (27.5%), and Schizophrenia/ Schizoaffective Disorder (27.5%). Other commonly reported disorders include generalized anxiety disorder, OCD, personality disorders and adjustment disorders. In addition, only 6.2% of participants reported having a diagnosis of PTSD.

**Table 1:** Demographic/clinical characteristics.

	**Study 1 (*****N*** **=** **536)**		**Study 2 (*****N*** **=127)**	
	***N***	**%**	***N***	**%**
Gender				
Male	288	53.7	50	39.4
Female	248	46.3	77	60.6
Race/ethnicity				
African American	248	46.3	55	43.3
White (non-Hispanic)	187	34.9	60	47.2
Hispanic	38	7.1	6	4.7
Other	26	4.9	6	4.7
Missing	37	6.9	0	0
Primary psychiatric diagnosis				
Schizophrenia/schizoaffective	92	27.5^*^	32	25.2
Depressive disorders	114	34.1^*^	42	33.1
Bipolar disorders	92	27.5^*^	48	37.8
Other	36	10.8^*^	5	3.9
Current psychotropic medication				
Antipsychotic			63	49.6
Mood stabilizer			30	23.6
Antidepressant			71	55.9
Anxiolytic/sedative			42	33.1
No medication			6	4.7
Disability benefits			55	43.3
	***M***	**SD**	***M***	**SD**
Age	47.23	12.91	46.41	11.97
Age at 1st hospitalization			26.65	12.57
Total # of hospitalizations			8.13	13.45
Total months hospitalized			14.64	47.68
BAI			23.64	11.98
BDI			27.22	11.85
CAPS-5			37.42	10.23
PCL-5	36.44	21.31	49.23	14.55
BPRS			47.42	8.45

*For Study 1, only 334 participants provided self-reported diagnosis*;

* *denotes valid percentages*.

In Study 2, 127 participants completed the baseline interview (see Table 1). Participants were typically in their late 40s (*M* = 45.97, SD = 11.94), mostly female (61.4%), and were nearly evenly split between African-American (42.4%) and white (43.9%) racial groups.

### Measures

#### Study 1

In Study 1, an abbreviated 16-item version of the *Traumatic Life Events Questionnaire* (TLEQ) (34) was used to screen lifetime trauma history for all participants. For each event on the scale, the participant indicated whether they had ever experienced it over their lifetime in a binary (yes/no) format. The TLEQ asks about the experience of traumatic events using wording that corresponds with the DSM-IV criterion A for PTSD. This version of the TLEQ was used to screen for trauma exposure in previous studies with persons with SMI (35).

The *PTSD checklist for DSM-5* (PCL-5) was used to assess the severity of the participant's symptoms (the PCL-5 was also administered in the baseline interview). The PCL-5 is a 20-item self-report measure that assesses the 20 DSM-5 symptoms of PTSD (36). This assessment can be used to screen individuals for PTSD and to make a provisional PTSD diagnosis. The wording of the PCL-5 items reflects both changes to existing symptoms and the addition of new symptoms in DSM-5. The self-report rating scale is 0–4 for each symptom, reflecting descriptors ranging from “Not at all,” to “Extremely.” The PCL-5 was administered without Criterion A since trauma exposure was measured previously by TLEQ. A total symptom severity score (range 0–80) can be obtained by summing the scores for each of the 20 items. A provisional PTSD diagnosis can be made by treating each item rated as 2 = “Moderately” or higher as a symptom endorsed, then following the DSM-5 diagnostic rule which requires at least: 1 B item (intrusion questions 1–5), 1 C item (avoidance questions 6–7), 2 D items (negative cognitions/affect questions 8–14), 2 E items (hyperarousal questions 15–20). Preliminary work suggests that a PCL-5 cut-off of 33 indicates probable PTSD. Subsequent analyses applied the following criteria to calculate provisional and probable PTSD: (1) total PCL-5 score ≥ 33, suggesting probable PTSD (National Center for PTSD, n.d.); and (2) Using B- E criteria of the DSM-5, suggesting provisional PTSD (National Center for PTSD, n.d.). A case meeting both conditions were considered as having PTSD.

The *Prolonged Grief-13* (PG-13) (24) was used to screen for Prolonged Grief Disorder (PGD) among participants who reported experiencing death of a loved one. PGD a newly recognized psychiatric disorder in the International Classification of Diseases, 11th Revision, and the DSM-5 text revision (37). As of this writing, the DSM-5 Text Revision Steering Committee formally approved PGD for inclusion as a new mental disorder (38). The PG-13 was developed to assess severity of grief reactions. Participants are asked to report the frequency (1–5) with which they experience each of the two questions assessing separation distress (yearning and intense emotional pain). The third question assessed if yearning or intense emotional pain were experienced daily 6 months' post-loss. The rest of the 8 items assessed the frequency or severity of feelings, thoughts, or actions associated with the loss of a loved one. Item 13 assesses significant reduction in social, occupational, or other important areas of functioning (yes or no). A positive screen for PGD diagnosis requires participants to meet the following criteria: (1) having experienced bereavement (i.e., significant loss), (2) the presence of separation distress at 6-months post-loss or beyond as indicated by a score of ≥4 on symptom severity of yearning, (3) a score of 4 or greater for 5 of 9 cognitive/emotional/behavioral symptoms and (4) endorsing significant functional impairment. The PG-13 has good psychometric properties that identify bereaved persons at heightened risk for enduring distress and dysfunction. In this study, we focused on PGD symptoms and used first 12 items from PG-13, omitting the last item on impairment. Those who met criteria 1–3 were considered as having probable PGD.

#### Study 2

In Study 2, participants who scored >33 on PCL-5 and were willing to participate in the larger intervention study were approached for a full baseline interview (*n* = 132). The Clinician Administered PTSD Scale for DSM-5 [(39); CAPS-5] is the “gold standard” in PTSD assessment, and was administered to assess PTSD diagnosis and severity of PTSD symptoms. The CAPS-5 is a 30-item structured interview that corresponds to the DSM-5 criteria for PTSD. Clinician Administered PTSD Interviews have been used to diagnose with persons with SMIs in various clinical studies (35). Depression was assessed with the Beck Depression Inventory-II [BDI-II; (40)] and general psychiatry symptoms with the Expanded Brief Psychiatric Rating Scale [BPRS; (41)]. Several aspects of social functioning, including activities of daily life, participation in work, social activity and family contact, as well as subjective life satisfaction, were assessed with the Brief Quality of Life Interview [QOLI; (42)]. The average of subjective indicators for quality of life was calculated, including general life satisfaction, and satisfaction with: living situation, daily activity, family contact, social relations, finances, job satisfaction, safety, and health. Ratings ranged from “terrible” to “delighted” on a 1–7 scale. Past employment history was collected by self-report as part of the demographic portion of the interview. Past employment history was defined by how many months the individual worked in a competitive employment setting for the past 5 years. This interval was chosen because it has been used in previous research (43). Wages earned in the past 6 months, hours worked during the 6 and 12-month intervals were entered as continuous variables.

### Data Analysis

Data were entered and cleaned using SPSS 26. Missing data were handled using list-wise deletion for the TLEQ questionnaires. For PCL-5, only two participants had completed less than half of the PCL-5 items, and 473 (88.6%) had complete data on the PCL-5. Missing data on PCL-5 were handled through mean imputation. Chi-square, *t*-test, correlational and regression analyses were then conducted. In Study 1, descriptive statistics were used to assess the rate of both PTSD and PGD. Gender differences of trauma exposure were analyzed with Chi-square analyses and *t*-test. Regression was used to evaluate predictors of PTSD severity. In Study 2, Chi-square and *t*-test were also used to analyze the different clinical outcomes between the group of individuals who had PGD and PTSD vs. PTSD only.

## Results

### Study 1

#### Trauma Exposure in Participants

The sample in Study 1 consisted of 536 participants (Table 1). The vast majority of participants were exposed to trauma, with 92.4% (*n* = 495 out of 536) experiencing at least one traumatic event, with large percentages of participants experiencing multiple types of traumas including physical and sexual assault. Table 2 lists different types of traumatic events reported by participants using the TLEQ, separated by gender. Both genders reported having their life threatened (53.2%), witnessing domestic violence during childhood (49.6%), domestic violence (47.1%), assault by stranger (41.5%), childhood physical abuse (34.3%), childhood sexual abuse by adult (35.1%), childhood sexual abuse by peer (28.6%), adult sexual abuse (26.1%) at high frequencies. Males reported exposure to warfare at higher frequency than female study participants (16.1% vs. 4.9%). Female participants reported higher frequencies than males for childhood sexual abuse by adult (48.4% vs. 23.7%), childhood sexual abuse by peer (39.3% vs. 19.4%), adult sexual abuse (38.3% vs. 15.4%), being stalked (45.2% vs. 31.5%), domestic violence (59.3% vs. 36.6%, and witnessing domestic violence (56.9% vs. 43.4%).

**Table 2:** Events reported on traumatic life events questionnaire by gender for Study 1 (*N* = 536).

**Life event**	**Total (*****N*** **=** **536)**		**Male (*****N*** **=** **288)**		**Female (*****N*** **=** **248)**		***x* ^**2**^**	***P***
	***N***	**Valid %**	**N**	**Valid %**	***N***	**Valid %**		
Car accident	138	25.7	69	24.0	69	27.8	1.04	0.31
Other accident	122	22.9	72	25.0	50	20.4	1.58	0.21
Warfare/combat	58	10.9	46	16.1	12	4.9	17.22	0.000
Sudden death	363	68.0	190	66.0	173	70.3	1.16	0.28
Robbery with weapon	197	36.8	114	39.6	83	33.5	2.14	0.14
Stranger assault	222	41.5	126	43.9	96	38.7	1.48	0.22
Witnessed stranger assault	215	40.1	127	44.1	88	35.5	4.12	0.04
Threatened death or injury	282	53.2	143	50.5	139	56.3	1.75	0.19
Childhood Physical Abuse	183	34.3	94	32.8	89	36.2	0.69	0.41
Witnessed domestic violence in childhood	264	49.6	124	43.4	140	56.9	9.72	0.002
Domestic Violence	252	47.1	105	36.6	147	59.3	27.49	0.000
Childhood sexual abuse by adult	188	35.1	68	23.7	120	48.4	35.59	0.000
Childhood sexual abuse by peer	153	28.6	56	19.4	97	39.3	25.60	0.000
Adult sexual abuse	139	26.1	44	15.4	95	38.3	35.97	0.000
Being stalked	202	37.8	90	31.5	112	45.2	10.59	0.001
Other distressing events	202	37.7	115	39.9	87	35.1	1.34	0.25
	**M** **±SD**^**a**^		**M** **±SD**^**b**^		**M** **±SD**^**c**^		***t***	***p***
Total life events	5.87 ± 4.03		5.44 ± 4.16		6.37 ± 3.82		−2.62	0.01

a *n = 511*;

b *n = 275*;

c *n = 236. Missing data for all three groups was handled by list-wise deletion*.

Table 3 lists the traumatic events identified by participants as most distressing on the TLEQ. One hundred and five participants did not specify the most distressing event, resulting in 431 participants providing data on most distressing events. The most frequently endorsed most distressing event, across gender, was the sudden death of a loved one/friend (26.5%), followed by childhood sexual abuse by an older person (8.8%) and domestic violence (7.2%). Among women, the most commonly reported most distressing events were sudden death of a loved one (24.9%), childhood sexual abuse by an older person (14.1%), and being a victim of domestic violence (11.7%). Among men, the most common most distressing events were the sudden death of a loved one (27.9%), stranger assault (8.8%), childhood sexual abuse by someone 5 years older (4.0%), car accidents (4.0%) and witnessing domestic violence (4.0%). On average and across genders, the total number of types of traumatic events experienced by participants was 5.87 on average; moreover, trauma reported as most distressing had occurred almost 20 years prior to the screening (18.02 ± 15.67).

**Table 3:** Traumatic events identified as most distressing by gender for Study 1 (*N* = 536).

	**Total (*****N*** **=** **431)**		**Male (*****N** **=*** **226)**		**Female (*****N** **=*** **205)**	
	***N***	**%**	***N***	**%**	***N***	**%**
Car accidents	14	3.2	9	4.0	5	2.4
Other accidents	11	2.6	7	3.1	4	2.0
Warfare	8	1.9	6	2.7	2	1.0
Sudden death of loved one	114	26.5	63	27.9	51	24.9
Robbery	14	3.2	8	3.5	6	2.9
Stranger assault	30	7.0	20	8.8	10	4.9
Witnessing violence	7	1.6	6	2.7	1	0.5
Threatened death or injury	11	2.6	7	3.1	4	2.0
Childhood physical abuse	10	2.3	7	3.1	3	1.5
Witnessing domestic violence	17	3.9	9	4.0	8	3.9
Domestic violence	31	7.2	7	3.1	24	11.7
Childhood sexual abuse by adult	38	8.8	9	4.0	29	14.1
Childhood sexual abuse by peer	6	1.4	1	0.4	5	2.4
Adult sexual abuse	20	4.6	6	2.7	14	6.8
Been stalked	7	1.6	4	1.8	3	1.5
Other	93	21.6	57	25.2	36	17.6
	***M***	**SD**	***M***	**SD**	***M***	**SD**
Years since most distressing event Age during index trauma	18.02 29.18	15.67 15.89	17.90 30.38	15.08 15.74	18.14 27.80	16.36 15.99

*105 cases out of 536 cases were excluded due to non-specifying the most distressing event, resulting in 431 total cases*.

#### Rate of Probable and Provisional PTSD in Participants

Two hundred sixty-five out of 536 (49.6%) participants met criteria for probable and provisional PTSD: (1) PCL-5 ≥ 33 and (2) PTSD criteria B-E met. PCL total score was moderately correlated with the overall number of types of trauma exposed to as reported in the TLEQ (*r* = 0.54, *p* < 0.01). Specific traumatic experiences (with the exception of warfare) were also significantly correlated with PTSD symptom severity.

#### Rate of Probable Prolonged Grief Disorder in Participants

Out of the sample of 536 screened, 407 completed the PG-13. Findings revealed the following: (1) 79 out of 536 (14.7%) met criteria for probable PGD; (2) 64 (11.9%) met criteria for both probable PGD and probable and provisional PTSD based on (1) total PCL-5 ≥ 33 and (2) B-E criteria. Among people who had PTSD based on (1) total PCL-5 ≥ 33 and (2) B-E criteria, the odds of meeting the criteria for PTSD were nearly 5 times greater among those with PGD (OR = 4.94, 2.70–9.03 for 95% confidence intervals; *p* < 0.0001) than among those without. The PTSD with probable PGD group had significantly higher PCL-5 scores (59.71 ± 12.70) than the PTSD without probable PGD group.

Study 2 examined clinical correlates of those who had both PTSD and probable PGD. Among the Study 2 participants, of the 127 who were found to meet CAPS criteria for PTSD, 25 (20.2%) were found to also meet criteria for probable PGD using the PG-13. The PTSD with probable PGD group were more likely to be African American than non-African American participants (*P* < 0.05). Moreover, the PTSD with probable PGD had more significant PCL-5 scores [56.00 ± 15.78] than the PTSD without probable PGD group (47.54 ± 14.13; Table 4). Regarding symptoms and quality of life, participants with and without probable PGD did not differ on the CAPS-5, BAI, BDI-II, BPRS, or QOLI measures. However, PTSD participants with probable PGD differed significantly from participants without probable PGD in work histories. Notably, the PTSD with probable PGD group had worked on average 36 months less at their longest held job (39.8 ± 32.2) than the PTSD without probable PGD group (74.45 ± 67.64). Participants in PTSD with probable PGD group did not differ from the PTSD without PGD group for total number of months of non-competitive jobs, sheltered work, or the total number of months of a competitive job in the past year (see Table 4).

**Table 4:** *T*-Test table for Study 2 (*N* = 127).

	**Total Sample (*****N** **=*** **127)**		**PTSD with PGD (*****N** **=*** **25)**		**PTSD without PGD (*****N** **=*** **102)**		***x* ^2^**	***p***
	**N**	**%**	**N**	**%**	**N**	**%**		
Gender							0.71	0.40
Male	50	39.4	8	32.0	42	41.2		
Female	77	60.6	17	68.0	60	58.8		
Race/ ethnicity							15.29	0.00
African american	55	43.3	17	68.0	38	37.3		
White (non-Hispanic)	60	47.2	5	20.0	55	53.9		
Hispanic	6	4.7	2	8.0	4	3.9		
Other	6	4.7	1	4.0	5	4.9		
Racial group analysis							7.73	0.01
African American	55	43.3	17	68.0	38	37.3		
Non-African American	72	56.7	8	32.0	64	62.7		
Primary psychiatric diagnosis							3.04	0.55
Schizophrenia/ schizoaffective	32	25.2	8	32.0	24	23.5		
Depressive disorders	42	33.1	10	40.0	32	31.4		
Bipolar disorders	48	37.8	7	28.0	41	40.2		
Other	5	3.9	0	0.0	5	4.9		
	***M***	**SD**	***M***	**SD**	***M***	**SD**	***t***	***p***
Age	46.41	11.97	45.07	12.07	46.74	11.98	0.62	0.54
BAI	23.64	11.98	24.79	15.27	23.35	11.10	−0.54	0.59
BDI	27.22	11.85	29.84	13.39	26.58	11.43	−1.24	0.22
CAPS-5	37.42	10.23	39.68	12.32	36.86	9.64	−1.24	0.22
PCL-5	49.23	14.55	56.00	15.78	47.57	13.82	−2.66	0.01
BPRS	47.42	8.45	46.70	8.01	47.60	8.58	0.48	0.63
Quality of life subjective average	3.70	0.94	3.64	0.95	3.71	0.95	−0.33	0.75
**Employment history**								
Total time at longest job in months?	66.32	62.8	39.8	32.2	74.45	67.64	−2.37	0.02
Total time at last job in months?	24.91	34.12	21.67	29.56	25.99	35.64	−0.52	0.60
How many hours per week at last job?	23.23	16.71	22.63	14.10	23.42	17.53	−0.20	0.85
Hourly wage at last job	12.92	9.00	12.07	6.95	13.18	9.56	−0.51	0.61
Total months had competitive job in past 5 years?	20.32	22.84	18.13	30.38	20.96	20.33	−0.52	0.60
Total months had non-competitive job in past 5 years?	1.46	4.67	2.5	6.80	1.14	3.78	1.25	0.22
Total months had non-shelter job in past 5 years?	0.06	0.60	0.00	0.00	0.08	0.69	−0.55	0.59
Total months working competitive job in last year	6.06	11.50	4.52	6.07	6.52	12.66	−0.73	0.47
how many months working any paid job last year	6.02	10.13	5.67	6.26	6.14	11.09	−0.20	0.84
Age at first hospitalization	25.58	13.40	23.74	13.04	26.01	13.52	−0.66	0.51
Number of times hospitalized	8.13	13.45	9.52	14.30	7.79	13.30	0.53	0.60
Total months hospitalized	14.64	47.68	8.84	16.92	16.99	55.51	−0.63	0.53
Days of alcohol use in past 30 days	0.91	2.84	1.67	3.90	0.72	2.50	1.47	0.15
Days gotten drunk in past 30 days	0.17	0.539	0.05	0.22	0.20	0.59	−1.18	0.24

## Discussion

This is the first study, to our knowledge, to examine the rates and correlates of PTSD and probable PGD among SMI clients. The findings are generally consistent with previous findings in indicating that trauma exposure is highly prevalent among persons with SMIs (9). Compared to other studies that used the TLEQ to assess trauma exposure in different populations (10, 39, 44), participants in the current study had higher rates of witnessing family violence and childhood sexual abuse but similar or lower levels of exposure to other types of traumatic events listed on TLEQ compared to 129 clients receiving opioid dependence treatment. Participants in this study reported higher rates of domestic violence, adult sexual assault, and childhood sexual abuse but reported lower rates of exposure to other types of trauma such as warfare and stranger assault compared to 207 male military veterans self-identified as having PTSD (39). In addition, the direction of sex differences on trauma exposure is consistent with those found in the general population and in the population of persons with SMIs (10, 45). Participants in this study reported lower rates of trauma exposure to each category of trauma listed on TLEQ compared to CMHC clients pre-selected for probable PTSD in Lu et al. (10). The finding is consistent with the fact that this study utilized trauma screening applied to all interested participants receiving vocational services at CMHCs, and did not preselect those with PTSD. The total trauma exposure reported in this study was around six types of trauma events which was significantly lower than seven types among CMHC participants preselected with probable PTSD in Lu et al. (10), and nine types among military veterans with self-identified PTSD described by Weathers et al. (39). Also, time interval since the most distressing trauma was approximately 18 years ago, similar to that of CMHC participants with probable PTSD described by Lu et al. (10), indicating that the most distressing event typically happened early on in life.

Regarding the relationship between PTSD and PGD, we found that nearly half of participants met criteria for PTSD, while roughly 15% met criteria for probable PGD, whereas, roughly 12% met the criteria for both PTSD and probable PGD. Further, having probable PGD increased the odds of having PTSD by nearly 5 times. The combination of PTSD and probable PGD also appeared to have a clinical impact, as participants who met criteria for PTSD and probable PGD had higher PTSD symptom severity. This is consistent with previous findings among veterans with combat-related PTSD that those with PGD had greater severity of PTSD (46). SMI clients who have experienced grief and loss may experience particular challenges that may require additional support. O'Hare and Sherrer (47) suggest that clients with SMIs may benefit from grief counseling; research is needed for such an intervention for this population. Clients with both PTSD and PGD present as a group with a higher service need, which is consistent with the conceptualization of multimorbidity in recent literature (28, 29). While there are some evidence-based practices for PGD (48, 49), there has not been efficacy studies on treatment of traumatic loss (50), future efficacy studies are needed which integrate both PTSD and grief treatment interventions to provide trauma-informed services for SMI clients among whom, multi-morbidity is common. The finding that being African American was associated with increased likelihood of having both PTSD and probable PGD is consistent with the social and cultural determinants of traumatic loss/grief (51), in which determinants such as cultural, social factors related to loss may facilitate or complicate the grieving process and therefore call for culturally sensitive care for traumatic grief.

## Limitations

Some limitations of the present study should be noted. Diagnoses of PTSD in Study 1 were based on self-report and therefore may be less reliable than research-based diagnoses conducted using CAPS-5. Furthermore, the last item in PG-13 was omitted, lending less certainty to the reliability of the diagnosis of PGD. Research has indicated that using a measure such as Inventory of Complicated Grief (52) may result in a higher percentage of people meeting criteria for PGD than using PG-13 (53, 54). In study 1, diagnoses of PTSD and PGD were based on self-report. PGD was only assessed in participants indicating “sudden loss of a loved one”, thus participants with PGD following non-sudden death of a loved one were not assessed for PGD, possibly leading to under-estimation of PGD. Furthermore, because TLEQ data were collected in a dichotomous format, while it provided a sense of the range of exposure to different types of trauma which participants experienced, the number of traumatic experiences which participants had was undetermined (e.g., repeated experience of multiple traumatic events of the same type), thus precluding analysis of dose-response relationships between trauma load and PTSD severity. Moreover, the TLEQ did not distinguish sudden death from violent or accidental death. Therefore, those who reported their most distressing event as sudden death of loved ones might not have qualified for a criterion A event under the DSM-5, thus enabling a probable PTSD diagnosis made following sudden, non-violent death. Additionally, it is plausible that participants self-selected into the study possibly based on prior trauma, leading to an overestimate of the rate of trauma related disorders.

Future research needs to be conducted to more accurately assess the relationship between psychiatric symptoms and clinical outcomes for SMI clients with both PTSD and PGD. Future studies need to specify the types of sudden death as accidental or violent in the TLEQ. Further, participants were limited to those receiving vocational services at the community mental health agencies, therefore findings may not be generalizable to all SMI clients. A 2014 survey of mental health facilities serving clients with SMI reported that vocational services were offered at 20% of community mental health facilities in the US (55). PTSD and PGD therefore needs to be examined in a variety of clinical programs serving clients with SMI. In particular, PGD is now proposed for inclusion in DSM 5-TR which opens the door to new understandings of how trauma and loss collectively influence the course of SMI. As our findings suggest, many individuals living with the multi-morbidity of SMI, PTSD and PGD would benefit from specialized intervention.

## Data Availability Statement

The datasets presented in this article are not readily available because at the time of data collection (2014–2018), the informed consent did not include languages to allow participants to consent to having the data deposited to a data repository, so unfortunately, we will not be able to make the dataset available to the public. However after 2018, the funding agency NIDILRR has made a requirement for all funding projects to include languages in the consent form to allow the possibility of having the data made publicly available through data repository mechanisms. Requests to access the datasets should be directed to Weili Lu, luwe1@shp.rutgers.edu.

## Ethics Statement

The studies involving human participants were reviewed and approved by Newark Health Sciences Institutional Review Board. The patients/participants provided their written informed consent to participate in this study.

## Author Contributions

WL was the PI for this project, designed the project, supervised the operation of the project and data collection, data entry, data analysis, and contributed to the conception and the writing of the manuscript. PY was the co-investigator of the project, supervised the project, and contributed to the conceptualization and writing. WW was the co-PI for the project, supervised the project, and contributed to the writing. CG and CB contributed to the data collection and data analysis. GG was the coinvestigator of the project, supervised data collection, and also did data entry. KG was the co-investigator and contributed to data analysis and writing. DB contributed to data cleaning, data analysis, editorial assistance, and the composition of the tables. HP provided study instruments for PGD and contributed to conceptualization and writing. All authors contributed to the article and approved the submitted version.

## Funding

This research was funded by National Institute on Disability, Independent Living, and Rehabilitation Research [NIDILRR Grant #90IF0074 (formerly H133G140147)].

## Conflict of Interest

The authors declare that the research was conducted in the absence of any commercial or financial relationships that could be construed as a potential conflict of interest.

## Publisher's Note

All claims expressed in this article are solely those of the authors and do not necessarily represent those of their affiliated organizations, or those of the publisher, the editors and the reviewers. Any product that may be evaluated in this article, or claim that may be made by its manufacturer, is not guaranteed or endorsed by the publisher.

## Footnotes

^1^Lu W, Waynor W, Yanos PT, Parrott J, Gill K. *CBT for PTSD among persons enrolled in Supported Employment programs: Does therapy improve employment outcomes?* (unpublished).

## References

[1] Breslau N Peterson EL Poisson LM Schultz LR Lucia VC. Estimating post-traumatic stress disorder in the community: lifetime perspective and the impact of typical traumatic events. Psychol Med. (2004) 34:889–98. 10.1017/S0033291703001612 15500309

[2] Kessler RC Chiu WT Demler O Walters EE. Prevalence, severity, and comorbidity of 12-month DSM-IV disorders in the national comorbidity survey replication. Arch Gen Psychiatry. (2005) 62:617–27. 10.1001/archpsyc.62.6.617 15939839 PMC2847357

[3] Tolin DF Foa EB. Sex differences in trauma and posttraumatic stress disorder: a quantitative review of 25 years of research. Psychol Bull. (2006) 132:959–92. 10.1037/0033-2909.132.6.959 17073529

[4] Substance Abuse and Mental Health Services Administration (SAMHSA). Key Substance Use and Mental Health Indicators in the United States: Results from the 2017 National Survey on Drug Use and Health (HHS Publication No. SMA 18–5068, NSUDH Series H-53). Rockville, MD: Center for Behavioral Health Statistics and Quality, SAMHSA (2018).

[5] Álvarez M Roura P Osés A Foguet Q Solà J Arrufat F. Prevalence and clinical impact of childhood trauma in patients with severe mental disorders. J Nerv Ment Dis. (2011) 199:156–61. 10.1097/NMD.0b013e31820c751c 21346485

[6] Bendall S Jackson H Hulbert C McGorry P. Childhood trauma and psychotic disorders: A systematic, critical review of the evidence. Schizophr Bull. (2008) 34:568–79. 10.1093/schbul/sbm121 18003630 PMC2632421

[7] Khalifeh H Johnson S Howard LM Borschmann R Osborn D Dean K. Violent and non-violent crime against adults with severe mental illness. Br J Psychiatry J Mental Sci. 206:275–82. 10.1192/bjp.bp.114.147843 25698767

[8] Mauritz M Goossens P Draijer N van Achterberg T. Prevalence of interpersonal trauma exposure and trauma-related disorders in severe mental illness. Eur J Psychotraumatol. (2013) 4:19985. 10.3402/ejpt.v4i0.19985 23577228 PMC3621904

[9] Grubaugh AL Zinzow HM Paul L Egede LE Frueh BC. Trauma exposure and posttraumatic stress disorder in adults with severe mental illness: a critical review. Clin Psychol Rev. (2011) 31:883–99. 10.1016/j.cpr.2011.04.003 21596012 PMC3148295

[10] Lu W Yanos PT Silverstein SM Mueser KT Rosenberg SD Gottlieb JD. Public mental health clients with severe mental illness and probable posttraumatic stress disorder: trauma exposure and correlates of symptom severity. J Trauma Stress. (2013) 26:266–73. 10.1002/jts.21791 23508645 PMC3888861

[11] O'Hare T Sherrer M. Impact of the most frequently reported traumatic events on community mental health participants. J Hum Behav Soc Environ. (2009) 19:186–95. 10.1080/10911350802687158

[12] O'Hare T Sherrer M. Lifetime trauma, subjective distress, substance use, and PTSD symptoms in people with severe mental illness: Comparisons among four diagnostic groups. Community Ment Health J. (2013) 49:728–32. 10.1007/s10597-013-9620-8 23812793

[13] Mueser KT Essock SM Haines M Wolfe R Xie H. Posttraumatic stress disorder, supported employment, and outcomes in people with severe mental illness. CNS Spectr. (2004) 9:913–25. 10.1017/S1092852900009779 15616477

[14] Seow L Ong C Mahesh M Sagayadevan V Shafie S Chong S. A systematic review on comorbid post-traumatic stress disorder in schizophrenia. Schizophr Res. (2016) 176:441–51. 10.1016/j.schres.2016.05.004 27230289

[15] Calhoun PS Bosworth HB Stechuchak KA Strauss JL Butterfield MI. The impact of posttraumatic stress disorder on quality of life and health service utilization among veterans who have schizophrenia. J Trauma Stress. (2006) 19:393–7. 10.1002/jts.20114 16789002

[16] Mansueto G Faravelli C. Stressful life events and psychosis gender differences. Stress Health. (2021). 10.1002/smi.3067. [Epub ahead of print]. 33973342

[17] Mansueto G Caselli G Ruggiero GM Sassaroli S. Metacognitive beliefs and childhood adversities: an overview of the literature. Psychol Health Med. (2019) 24:542–50. 10.1080/13548506.2018.1550258 30463429

[18] Mansueto G Schruers K Cosci F van Os J Alizadeh BZ Bartels-Velthuis AA. Childhood adversities and psychotic symptoms: the potential mediating or moderating role of neurocognition and social cognition. Schizophrenia Research. (2019) 206:183–93. 10.1016/j.schres.2018.11.028 30527930

[19] Struck N Krug A Yuksel D Stein F Schmitt S Meller T. Childhood maltreatment and adult mental disorders—the prevalence of different types of maltreatment and associations with age of onset and severity of symptoms. Psychiatry Res. (2020) 293:113398. 10.1016/j.psychres.2020.113398 32920524

[20] Kessler RC Aguilar-Gaxiola S Alonso J Benjet C Bromet EJ Cardoso G. Trauma and PTSD in the WHO World Mental Health Surveys. Eur J Psychotraumatol. (2017) 8:1353383–16. 10.1080/20008198.2017.1353383 29075426 PMC5632781

[21] Prigerson H Vanderwerker LC Maciejewski PK. A case for inclusion of prolonged grief disorder in DSM-V. In: Handbook of Bereavement Research and Practice: Advances in Theory and Intervention. p. 165–86 (2008). 10.1037/14498-008

[22] Goldsmith B Morrison RS Vanderwerker LC Prigerson HG. Elevated rates of prolonged grief disorder in African Americans. Death Stud. (2008) 32:352–65. 10.1080/07481180801929012 18850684

[23] Golden AM Dalgleish T. Is prolonged grief distinct from bereavement-related posttraumatic stress? Psychiatry Res. (2010) 178:336–41. 10.1016/j.psychres.2009.08.021 20493535

[24] Prigerson HG Horowitz MJ Jacobs SC Parkes CM Aslan M Goodkin K. Prolonged grief disorder: psychometric validation of criteria proposed for DSM-V and ICD-11. PLoS Med. (2009) 6:1–12. 10.1371/journal.pmed.1000121 19652695 PMC2711304

[25] Jacobs SC. Pathological Grief: Maladaptation to Loss. (1993). American Psychiatric Press.

[26] Maercker A Lalor J. Diagnostic and clinical considerations in prolonged grief disorder. Dialogues Clin Neurosci. (2012) 14:167–76. 10.31887/DCNS.2012.14.2/amaercker 22754289 PMC3384445

[27] Maciejewski PK Maercker A Boelen PA Prigerson HG. “Prolonged grief disorder” and “persistent complex bereavement disorder”, but not “complicated grief”, are one and the same diagnostic entity: An analysis of data from the yale bereavement study. World Psychiatry. (2016) 15:266–75. 10.1002/wps.20348 27717273 PMC5032512

[28] Bhalla IP Rosenheck RA. A change in perspective: from dual diagnosis to multimorbidity. Psychiatric Services. (2018) 69:112–6. 10.1176/appi.ps.201700194 29032702

[29] Thimm JC Kristoffersen AE Ringberg U. The prevalence of severe grief reactions after bereavement and their associations with mental health, physical health, and health service utilization: a population-based study. Eur J Psychotraumatol. (2020) 11:1844440. 10.1080/20008198.2020.1844440 33408813 PMC7748058

[30] Ward MC White DT Druss BG. A meta-review of lifestyle interventions for cardiovascular risk factors in the general medical population: lessons for individuals with serious mental illness. J Clin Psychiatry. (2015) 76:e477–86. 10.4088/JCP.13r08657 25919840

[31] Kristensen P Weisæth L Hussain A Heir T. Prevalence of psychiatric disorders and functional impairment after loss of a family member: a longitudinal study after the 2004 Tsunami. Depression Anxiety. (2015) 32:49–56. 10.1002/da.22269 24817217

[32] Burke LA Neimeyer RA McDevitt-Murphy ME. African American homicide bereavement: aspects of social support that predict complicated grief, PTSD, and depression. OMEGA—J Death Dying. (2010) 61:1–24. 10.2190/OM.61.1.a 20533646

[33] Craig CD Sossou M-A Schnak M Essex H. Complicated Grief and its relationship to mental health and well-being among Bosnian refugees after resettlement in the United States. Implications Pract Policy Res Traumatol. (2008) 14:103–15. 10.1177/1534765608322129

[34] Kubany ES Haynes SN Leisen MB Owens JA Kaplan AS Watson SB. Development and preliminary validation of a brief broad-spectrum measure of trauma exposure: The traumatic life events questionnaire. Psychol Assess. (2000) 12:210–24. 10.1037/1040-3590.12.2.210 10887767

[35] Mueser KT Gottlieb JD Xie H Lu W Yanos PT Rosenberg SD. Evaluation of cognitive restructuring for post-traumatic stress disorder in people with severe mental illness. Br J Psychiatry. (2015) 206:501–8. 10.1192/bjp.bp.114.147926 25858178 PMC4450219

[36] Weathers FW Litz BT Keane TM Palmieri PA Marx BP Schnurr PP. The PTSD Checklist for DSM-5 (PCL-5)—Standard. Available from https://www.ptsd.va.gov/ (2013).

[37] Killikelly C Maercker A. Prolonged grief disorder for ICD-11: The primacy of clinical utility and international applicability. Eur J Psychotraumatol. (2017) 8:1476441. 10.1080/20008198.2018.1476441 29887976 PMC5990943

[38] Lichtenthal WG Roberts KE Prigerson HG. Bereavement care in the wake of COVID-19: Offering condolences and referrals. Annals Internal Med. (2020). 10.7326/M20-2526 32574071 PMC7346775

[39] Weathers FW Bovin MJ Lee DJ Sloan DM Schnurr PP Kaloupek DG. The clinician-administered PTSD scale for DSM-5 (CAPS-5): Development and initial psychometric evaluation in military veterans. Psychol Assess. (2018) 30:383–95. 10.1037/pas0000486 28493729 PMC5805662

[40] Beck AT Steer RA Garbin MG. Psychometric properties of the beck depression inventory: twenty-five years of evaluation. Clin Psychol Rev. 8:77–100. 10.1016/0272-7358(88)90050-5

[41] Lukoff D Wallace CJ Liberman RP Burke K. A holistic program for chronic schizophrenic patients. Schizophrenia Bull. 12:274–82. 10.1093/schbul/12.2.274 3520804

[42] Lehman AF Kernan E DeForge BR Dixon L. Effects of homelessness on the quality of life of persons with severe mental illness. Psychiatric Serv. (1995) 46:922–26. 10.1176/ps.46.9.922 7583504

[43] Gao N Gill KJ Schmidt LT Pratt CW. The application of human capital theory in vocational rehabilitation for individuals with mental illness. J Vocational Rehabil. (2010) 32:25–33. 10.3233/JVR-2010-0492

[44] Peirce JM Burke CK Stoller KB Neufeld KJ Brooner RK. Assessing traumatic event exposure: comparing the traumatic life events questionnaire to the structured clinical interview for DSM-IV. Psychol Assess. (2009) 21:210–8. 10.1037/a0015578 19485675 PMC3037764

[45] Breslau N Kessler RC Chilcoat HD Schultz LR Davis GC Andreski P. Trauma and posttraumatic stress disorder in the community: the 1996 detroit area survey of trauma. Arch Gen Psychiatry. (1998) 55:626–32. 10.1001/archpsyc.55.7.626 9672053

[46] Simon NM Hoeppner SS Lubin RE Robinaugh DJ Malgaroli M Norman SB. Understanding the impact of complicated grief on combat related posttraumatic stress disorder, guilt, suicide, and functional impairment in a clinical trial of post-9/11 service members and veterans. Depress Anxiety. (2020) 37:63–72. 10.1002/da.22911 31916660 PMC7433022

[47] O'Hare T Sherrer M. Subjective distress associated with sudden loss in clients with severe mental illness. Commun Mental Health J. 47:646–53. 10.1007/s10597-011-9382-0 21246273

[48] Shear K Reynolds CF Simon NM Zisook S Wang Y Mauro C. Optimizing treatment of complicated grief. JAMA Psychiatry. (2016) 73:685–94. 10.1001/jamapsychiatry.2016.0892 27276373 PMC5735848

[49] Johannsen M Damholdt MF Zachariae R Lundorff M Farver-Vestergaard I O'Connor M. Psychological interventions for grief in adults: a systematic review and meta-analysis of randomized controlled trials. J Affect Disorders. (2019) 253:69–86. 10.1016/j.jad.2019.04.065 31029856

[50] Smid GE Kleber RJ de la Rie SM Bos JB Gersons BP Boelen PA. Brief eclectic psychotherapy for traumatic grief (BEP-TG): Toward integrated treatment of symptoms related to traumatic loss. Eur J Psychotraumatol. (2015) 6:27324. 10.3402/ejpt.v6.27324 26154434 PMC4495623

[51] Smid GE. A framework of meaning attribution following loss. Eur J Psychotraumatol. (2020) 11:1776563. 10.1080/20008198.2020.1776563 33244357 PMC7678673

[52] Prigerson HG Maciejewski PK Reynolds CF Bierhals AJ Newsom JT Fasiczka A. Inventory of complicated grief: a scale to measure maladaptive symptoms of loss. Psychiatry Res. (1995) 59:65–79. 10.1016/0165-1781(95)02757-2 8771222

[53] Prigerson HG Maciejewski PK. Rebuilding consensus around valid criteria for disordered grief. JAMA Psychiatry. (2017) 74:435–6. 10.1001/jamapsychiatry.2017.0293 28355449 PMC5499154

[54] Maciejewski PK Prigerson HG. Prolonged, but not complicated, grief is a mental disorder. Br J Psychiatry. (2017) 211:189–91. 10.1192/bjp.bp.116.196238 28970298

[55] Sherman LJ Lynch SE Teich J Hudock W.J. Availability of supported employment in specialty mental health treatment facilities and facility characteristics: 2014. The CBHSQ Report (2017). 28749638

